# The effectiveness of transarterial chemoembolization in recurrent hepatocellular-cholangiocarcinoma after resection

**DOI:** 10.1371/journal.pone.0198138

**Published:** 2018-06-07

**Authors:** Seong Kyun Na, Gwang Hyeon Choi, Han Chu Lee, Yong Moon Shin, Jihyun An, Danbi Lee, Ju Hyun Shim, Kang Mo Kim, Young-Suk Lim, Young-Hwa Chung, Yung Sang Lee

**Affiliations:** 1 Department of Internal Medicine, Jeju National University Hospital, Jeju National University School of Medicine, Jeju, Korea; 2 Department of Gastroenterology, Asan Liver Center, Asan Medical Center, University of Ulsan College of Medicine, Seoul, Korea; 3 Department of Radiology and Research Institute of Radiology, Asan Medical Center, University of Ulsan College of Medicine, Seoul, Korea; Digestive Disease Research Center, Scott & White Healthcare, UNITED STATES

## Abstract

**Background:**

Combined hepatocellular-cholangiocarcinoma (cHCC-CC) can present as a hypervascular or peripherally enhancing tumor in dynamic imaging. We evaluated the effect of transarterial chemoembolization (TACE) on prognosis according to post-operative recurrence imaging patterns.

**Methods:**

We retrospectively analyzed 42 cHCC-CC and 59 hepatocellular carcinoma (HCC-control) patients at the Asan Medical Center. We classified recurrent cHCC-CC according to enhancement pattern (globally enhancing: GE cHCC-CC, peripherally enhancing: PE cHCC-CC) and evaluated tumor response, time-to-local progression (TTP_local_), and overall survival (OS).

**Results:**

The GE cHCC-CC group had a significantly higher best objective response rate (complete remission + partial response) than the PE cHCC-CC group (36% vs 0%, *P* = 0.005), and it was comparable to that of the HCC-control group (35.6%, *P* = 0.97). TTP_local_ in the GE cHCC-CC group was significantly shorter than in the HCC-control group (6.6 vs 27.1 months, *P* < 0.001), and was not significantly different from that in the PE cHCC-CC group (5.3 months, *P* = 0.12). OS was 12.4 months, 52.8 months, and 67.5 months in the PE cHCC-CC, GE cHCC-CC, and HCC-control groups, respectively (*P*s < 0.05). The adjusted hazard ratios (HRs) for TTP_local_ and OS revealed an independent association with enhancement pattern of recurrent cHCC-CC (TTP_local:_ HR 2.46; 95% CI 1.10–5.46; *P* = 0.03; OS: HR 5.97; 95% CI 2.38–14.96; *P* < 0.001).

**Conclusions:**

The GE cHCC-CC group showed better response and prognosis after TACE than the PE cHCC-CC group, but poorer response and prognosis than the HCC-control group. Enhancement patterns at recurrence were crucially associated with tumor response and overall survival.

## Introduction

Combined hepatocellular-cholangiocarcinoma (cHCC-CC) accounts for 0.4%–14.2% of primary liver malignancies [1, 2], and contains pathological components of both hepatocellular carcinoma (HCC) and cholangiocarcinoma (CC) [3]. Pre-operative diagnosis of cHCC-CC is sometimes challenging due to its heterogeneous imaging characteristics with overlapping features of both HCC and CC [4–7]. It can present predominantly as a hypervascular lesion resembling HCC or a peripherally enhancing lesion resembling CC, according to the predominant histologic component within the tumor. The enhancement patterns of cHCC-CC on dynamic computed tomography (CT) or magnetic resonance imaging (MRI) are known to be in good agreement with the dominant pathologic component [3, 8].

Surgical resection and liver transplantation are considered curative treatments for cHCC-CC. However, post-operative recurrence is frequent, and the 5-year survival rate after surgical resection is around 30% [9–13]. The prognosis after liver transplantation for cHCC-CC is poorer than that of HCC and confers a survival rate similar to that of selected patients with CC [14, 15].

Transarterial chemoembolization (TACE) has been used as a mainstay of palliative treatment for HCC [16, 17]. Tumor vascularity is a significant prognostic factor for TACE because chemotherapeutic agents and embolic material can be delivered much more effectively in hypervascular tumors. Many cHCC-CC tumors are less vascular and more fibrotic than HCC [2]; hence, the effectiveness of TACE for cHCC-CC is questionable. There have been no established treatments for recurrent cHCC-CC, although TACE can be considered as a palliative treatment. A previous study suggested that TACE response was highly related to tumor vascularity in patients with primary non-resectable cHCC-CC [18]. However, as far as we know, the therapeutic efficacy of TACE has not been systematically evaluated in recurrent cHCC-CC, especially according to enhancement patterns, although several studies have reported their relationship with patient prognosis [3, 18, 19].

The aim of the present study was to evaluate the therapeutic efficacy of TACE and patient prognosis according to the post-operative recurrence patterns of cHCC-CC in comparison with HCC.

## Patients and methods

The present study was reviewed and approved by the Institutional Review Board of the Asan Medical Center (IRB No. 2016–1125). Patient records and information were anonymized and de-identified prior to analysis.

### Patients

**Fig 1:**
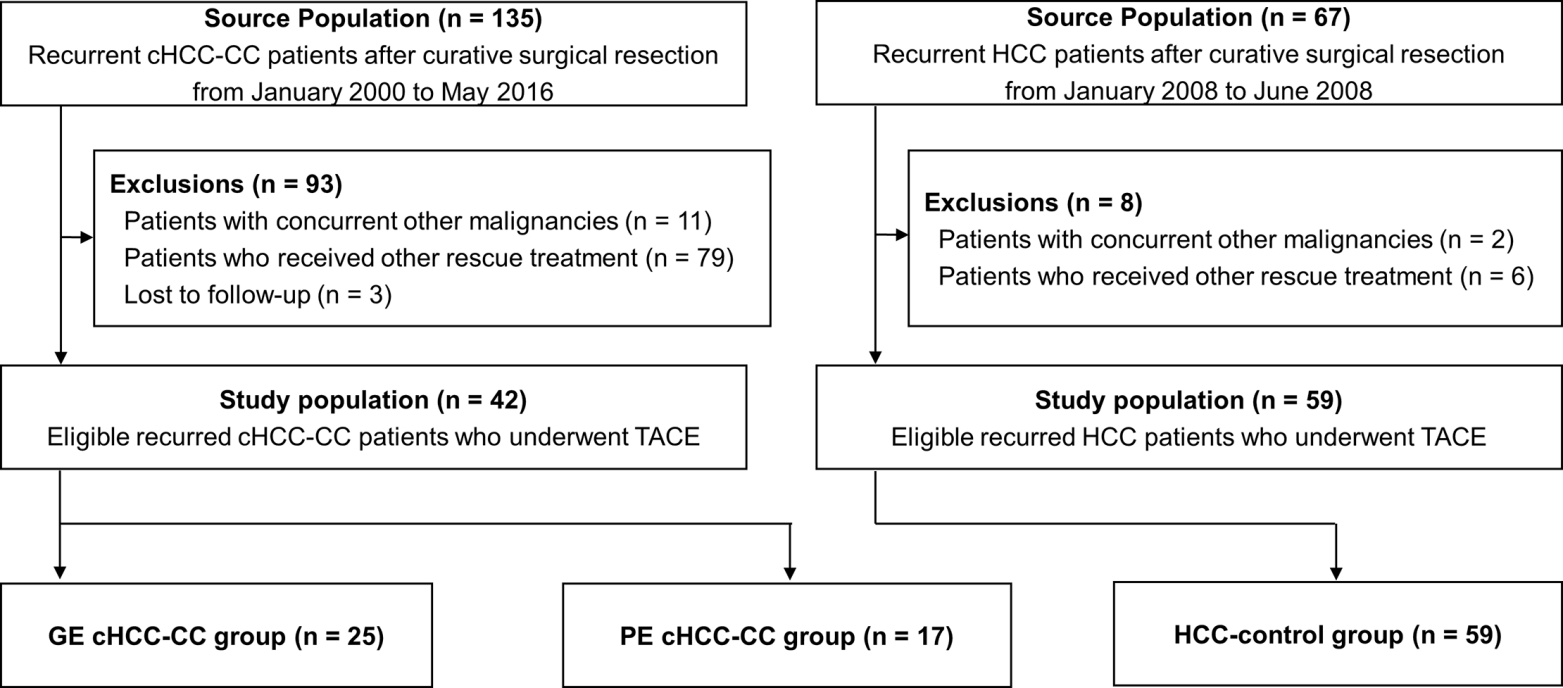
Patient flow diagram. cHCC-CC, combined hepatocellular-cholangiocarcinoma; GE, globally enhancing; PE, peripherally enhancing; TACE, transarterial chemoembolization; HCC, hepatocellular carcinoma.

Between January 2000 and May 2016, 287 patients who had undergone curative surgical resection at the Asan Medical Center were pathologically diagnosed as having cHCC-CC. Among these patients, recurrences occurred in 135 patients, and 93 patients were excluded for the following reasons: 11 patients had concurrent other malignancies; 3 patients were lost to follow-up; and 79 patients received treatments other than TACE for the first recurrence (surgical resection: 9, liver transplantation: 1, radiofrequency ablation: 11, percutaneous ethanol injection: 1, cytotoxic chemotherapy: 11, sorafenib: 20, radiation treatment: 8, and supportive treatment: 18). After exclusion, a total of 42 patients were analyzed retrospectively (Fig 1).

To compare treatment responses and prognosis, we also analyzed a total of 59 HCC patients treated with TACE for their first post-operative recurrence after curative surgical resection between January 2008 and June 2008. All patients received follow-up appointments every 2–3 months until death or 31 October 2016.

### Radiological evaluation

The dynamic CT/MRI findings, from diagnosis to end of follow-up, were analyzed by a radiologist (Y.M.S.) with 25 years of experience in imaging of the liver. We classified the tumors according to the enhancement patterns at the time of first recurrence after surgical resection. Tumors with global arterial enhancement (more than 50% of tumor volume) on CT/MRI were arbitrarily classified as recurrent globally enhancing (GE) cHCC-CC, while those that showed only peripheral/rim enhancement or iso-enhancement were classified as recurrent peripherally enhancing (PE) cHCC-CC.

Tumor response to TACE was evaluated with follow-up CT performed 4–6 weeks after TACE according to the Response Evaluation Criteria in Solid Tumors (RECIST) 1.1 criteria [20], since the viability of PE cHCC-CC after TACE could not be determined by enhancement criteria such as modified RECIST or the European Association for the Study of the Liver criteria [21, 22]. According to the RECIST criteria, complete remission (CR) was defined as the total disappearance of the lesion; partial response (PR) and progressive disease (PD) as a greater than 30% decrease and a greater than 20% increase, respectively, of the sum of the longest diameters of the target lesions; and stable disease (SD) as neither PR nor PD. The appearance of new lesions ≥ 1 cm in maximum diameter was defined as PD. A maximum of two index lesions in the liver were evaluated for time-to-progression (TTP) according to the RECIST 1.1 criteria [20]. To evaluate the therapeutic effectiveness of TACE, we also assessed the time-to-local progression (TTP_local_) during the follow-up period. TTP_local_ was defined as the time from the start of TACE to objective local progression of the initial recurrent hepatic mass, regardless of the appearance of a new lesion. We also assessed progression-free survival (PFS) and overall survival (OS) after recurrence.

### TACE protocol

The TACE protocol used in our institution has been described elsewhere [23]. All TACE sessions were performed by experienced radiologists. Both superior mesenteric and common hepatic arteriography were performed to assess overall anatomy, tumor burden, and portal vein patency. Cisplatin (Dong-A Pharmaceutical, Seosan, Korea) at 2 mg/kg was administered into the lobar hepatic artery for 15 minutes. After selective vascular catheterization of the tumor-feeding artery, an emulsion of 2–20 mL of iodized oil (Lipiodol Ultra-Fluide; Laboratoire Guerbet, Aulnay-Sous-Bois, France) and cisplatin in a 1:1 ratio was infused into the target arteries. The embolization of the arterial tumor feeders was then performed using 1-mm-diameter absorbable gelatin sponge particles (Gelfoam; Upjohn, Kalamazoo, MI, USA) until arterial flow stasis was achieved.

TACE was repeated every 2–3 months if residual viable tumor tissue was evident without the appearance of clinically significant extrahepatic metastases, main portal vein invasion, or deterioration in liver function. Though we applied the RECIST criteria for the evaluation of tumor response in this study, TACE was delayed until definite viable tumors were evident or new lesions appeared if there was dense lipiodol deposition and no definite tumor enhancement on dynamic CT scan.

### Statistical analysis

Statistical analysis was performed using the Student’s t-test or Mann-Whitney test for continuous variables, and the chi-square test or Fisher’s exact test for categorical variables. TTP, TTP_local_, PFS, and OS were calculated and plotted using the Kaplan-Meier method, and differences between the groups were analyzed using a log-rank test. Univariable and multivariable Cox proportional-hazard regression models were performed to identify prognostic factors in relation to tumor progression and survival. For the multivariable Cox proportional-hazard regression model, we included factors that were significant or that showed a trend toward significance (*P* < 0.1) in a univariable model and performed backward elimination (*P* < 0.05) using the “standard method” of removing the variable with the largest *P*-value. Potential for risk was expressed as a hazard ratio (HR) with corresponding 95% confidence interval (CI), and *P*-values less than 0.05 were considered to be statistically significant. All statistical analyzes were performed using the Statistical Package for the Social Sciences statistical software (version 21; SPSS-IBM, Chicago, IL, USA) or R version 3.02 (R Foundation for Statistical Computing, Vienna, Austria).

## Results

### Pre-operative baseline characteristics according to recurrence pattern

The baseline clinical and pathologic characteristics of GE cHCC-CC, PE cHCC-CC, and recurrent HCC control (HCC-control) patients before surgery are shown in Table 1. The mean ages of the GE cHCC-CC, PE cHCC-CC, and HCC-control groups were 54.2 ± 10.1, 52.6 ± 8.9, and 56.2 ± 9.8 years, respectively. All groups had a higher portion of male patients (GE cHCC-CC, 84%; PE cHCC-CC, 94.1%; and HCC-control, 89.8%). Chronic hepatitis B virus infection was the major etiologic factor (92%, 100%, and 84.7% in the GE cHCC-CC, PE cHCC-CC, and HCC-control groups, respectively), and about half of the patients had liver cirrhosis (56%, 47.1%, and 54.2%, respectively). Most of the patients had Child-Turcotte-Pugh class A (100%, 100% and 94.9% in the GE cHCC-CC, PE cHCC-CC, and HCC-control groups, respectively) and a single tumor at initial diagnosis (84%, 76.5%, and 76.3%, respectively). The median values of maximum tumor diameter were 3.5 (interquartile range [IQR], 2.2–9.3) cm in the GE cHCC-CC group, 5.5 (IQR, 3.6–7.5) cm in the PE cHCC-CC group, and 5.0 (IQR, 3.5–7.5) cm in the HCC-control group. Gross vascular invasion was observed in 12%, 5.9%, and 11.9% of the GE cHCC-CC, PE cHCC-CC, and HCC-control groups, respectively, and metastases to the lymph nodes or distant sites were not observed. Before operation, a GE pattern was observed in 14 of 25 (56%) patients in the GE cHCC-CC group and 4 of 17 (23.5%) patients in the PE cHCC-CC. Therefore, the agreement of enhancement patterns between the initial and recurrent tumor was 77.8% in the GE cHCC-CC group and 54.2% in the PE cHCC-CC group.

**Table 1:** Baseline characteristics of the study patients according to the recurrence pattern at the time of first recurrence.

	GE cHCC-CC (n = 25)	PE cHCC-CC (n = 17)	HCC-control (n = 59)	*P*^*a*^	*P*^*b*^
Age, years	54.2 ± 10.1	52.6 ± 8.9	56.2 ± 9.8	0.60	0.40
Male Gender	21 (84.0)	16 (94.1)	53 (89.8)	0.37	0.29
Aetiology				0.49	0.20
HBV	23 (92)	17 (100)	50 (84.7)		
HCV	0 (0)	0 (0)	4 (6.8)		
Alcohol	1 (4)	0 (0)	0 (0)		
Others	1 (4)	0 (0)	5 (8.5)		
Liver cirrhosis	14 (56)	8 (47.1)	32 (54.2)	0.57	0.88
Child-Pugh class A	25 (100)	17 (100)	56 (94.9)	> 0.99	0.55
Initial enhancement pattern				0.04	0.01
Global enhancement	14 (56)	4 (23.5)	49 (83.1)		
Peripheral enhancement	11 (44)	13 (76.5)	10 (16.9)		
Size, cm	3.5 (2.2–9.3)	5.5 (3.6–7.5)	5.0 (3.5–7.5)	0.88	0.90
Multiple tumors (≥2)	4 (16)	4 (23.5)	14 (23.7)	0.69	0.57
Gross vascular invasion	3 (12)	1 (5.9)	7 (11.9)	0.64	> 0.99
7^th^ AJCC stage				0.86	–
I	17 (68)	12 (70.6)	46 (73)		
II	8 (32)	5 (29.4)	6 (9.5)		
III	0 (0)	0 (0)	11 (17.5)		
Combined/mixed histology	24 (96) / 1 (4)	14 (82.4) / 3 (17.6)	–	0.29	–
AFP, ng/mL	36 (9.8–642.5)	9.7 (5.3–939.5)	50 (7.5–743)	0.40	0.31
CA 19–9, U/mL	18.4 (13.9–39.8)	25.2 (7.6–168.8)	14.4 (7.7–18.2)	0.82	0.03

Data presented as mean and standard deviation (SD) or median and IQR (interquartile range) or frequency (n) and percentage where appropriate.

a, between GE cHCC-CC and PE cHCC-CC

b, between GE cHCC-CC and HCC-control.

GE, globally enhancing; cHCC-CC, combined hepatocellular-cholangiocarcinoma; PE, peripherally enhancing; HBV, hepatitis B virus; HCV, hepatitis C virus; AJCC, American Joint Committee on Cancer system; AFP, α-feto protein; CA, carbohydrate antigen.

The major pathologic type of both GE cHCC-CC and PE cHCC-CC was combined (96% vs 82.4%, *P* = 0.29). Tumor staging according to the 7th American Joint Committee on Cancer system identified 17 (68%) stage I and 8 (32%) stage II cases in the GE cHCC-CC group and 12 (70.6%) stage I and 5 (29.4%) stage II cases in the PE cHCC-CC group.

### Clinical characteristics of the GE cHCC-CC, PE cHCC-CC, and HCC Groups at first recurrence

At the time of first recurrence, the mean ages of the GE cHCC-CC, PE cHCC-CC, and HCC-control groups were 55.1 ± 10.2, 53.2 ± 8.8, and 58.3 ± 9.6 years, respectively (Table 2). The median interval from resection to recurrence was significantly shorter in the GE cHCC-CC group than in the HCC-control group (5.2 vs. 16.4 months, *P* < 0.001), and was not significantly different between the GE cHCC-CC and PE cHCC-CC groups (5.2 vs. 5.9 months, *P* = 0.67). There was no significant difference in age, time to recurrence, proportion of single recurrence, tumor size, or the presence of portal vein invasion or metastasis between the GE cHCC-CC and PE cHCC-CC groups. Multiple recurrence and vascular invasion were significantly more frequent in the GE cHCC-CC group than in the HCC-control group (68% vs 32.2%, *P* = 0.002 and 16% vs 1.7%, *P* = 0.03, respectively), and there was no significant difference in distant metastasis (20% vs 6.8%, *P* = 0.12).

**Table 2:** Clinical characteristics of the study patients at the time of first recurrence.

	GE cHCC-CC (n = 25)	PE cHCC-CC (n = 17)	HCC-control (n = 59)	*P*^*a*^	*P*^*b*^
Time to recurrence, months	5.2 (3.1–10.4)	5.9 (3.2–9.9)	16.4 (6.1–40.7)	0.67	< 0.001
Age, years	55.1 ± 10.2	53.2 ± 8.8	58.3 ± 9.6	0.55	0.18
Child-Pugh class A	24 (96)	16 (94.1)	57 (96.6)	> 0.99	> 0.99
Size, cm	1.5 (1.2–2.3)	2.0 (1.1–3.4)	1.6 (1.1–2.3)	0.60	0.91
Multiple tumors (≥ 2)	17 (68)	12 (70.6)	19 (32.2)	> 0.99	0.002
Gross vascular invasion	4 (16)	0 (0)	1 (1.7)	0.13	0.03
Distant metastasis	5 (20)	5 (29.4)	4 (6.8)	0.71	0.12
AFP, ng/mL	7.1 (2.8–243)	5.4 (2.9–49)	7.9 (2.4–42.7)	0.35	0.50

Data presented as mean and standard deviation (SD) or median and IQR (interquartile range) or frequency (n) and percentage where appropriate.

a, between GE cHCC-CC and PE cHCC-CC

b, between GE cHCC-CC and HCC-control.

GE, globally enhancing; cHCC-CC, combined hepatocellular-cholangiocarcinoma; PE, peripherally enhancing; AFP, α-feto protein.

### Tumor response outcome to TACE

A median of 2 (range, 1–4) cycles of TACE were performed in the cHCC-CC group and 5 (range, 2–8) cycles were performed in the HCC-control group (*P* = 0.002). Tumor response was evaluated 4–6 weeks after the first cycle of TACE by RECIST criteria. At the first evaluation, 41.2% of patients in the PE cHCC-CC group had PD, while 20% and 15.3% in the GE cHCC-CC and HCC-control groups had PD, respectively (Table 3). The objective response rate (CR plus PR) and the disease control rate (CR plus PR plus SD) in the GE cHCC-CC and PE cHCC-CC groups were not significantly different (12% vs. 0%, *P* = 0.26 and 80% vs. 58.8%, *P* = 0.17, respectively). Moreover, the objective response rate and disease control rate in the GE cHCC-CC and HCC-control groups were comparable (12% vs. 8.5%, *P* = 0.69 and 80% vs. 84.7%, *P* = 0.75, respectively). However, the disease control rate in the PE cHCC-CC and HCC-control groups was significantly different (58.8% vs. 84.7%, *P* = 0.02).

**Table 3:** Post-TACE response and best response according to the first recurrence patterns using RECIST criteria.

Variables	GE cHCC-CC (n = 25)	PE cHCC-CC (n = 17)	HCC-control (n = 59)	*P*^*a*^	*P*^*b*^	*P*^*c*^
Post TACE response				0.15	0.73	0.045
Complete remission	0 (0)	0 (0)	0 (0)			
Partial response	3 (12)	0 (0)	5 (8.5)			
Stable disease	17 (68)	10 (58.8)	45 (76.2)			
Progressive disease	5 (20)	7 (41.2)	9 (15.3)			
Objective response rate^†^	3 (12)	0 (0)	5 (8.5)	0.26	0.69	0.58
Disease control rate^‡^	21 (80)	10 (58.8)	50 (84.7)	0.17	0.75	0.02
Best response				0.04	0.39	0.01
Complete remission	2 (8)	0 (0)	11 (18.7)			
Partial response	7 (28)	0 (0)	10 (16.9)			
Stable disease	11 (44)	10 (58.8)	30 (50.8)			
Progressive disease	5 (20)	7 (41.2)	8 (13.6)			
Objective response rate ^†^	9 (36)	0 (0)	21 (35.6)	0.005	0.97	0.004
Disease control rate^‡^	20 (80)	10 (58.8)	51 (86.4)	0.17	0.52	0.01

Data presented as frequency (n) and percentage.

a, between GE cHCC-CC and PE cHCC-CC

b, between GE cHCC-CC and HCC-control

c, between PE cHCC-CC and HCC-control

† Complete remission + partial response

‡ Complete remission + partial response + stable disease

The best tumor response was significantly different according to enhancing pattern at recurrence. The objective response rates were 0% in the PE cHCC-CC group, 36.0% in the GE cHCC-CC group, and 35.6% in the HCC-control group (*P* = 0.005 for PE cHCC-CC vs. GE cHCC-CC; *P* = 0.97 for GE cHCC-CC vs. HCC-control). The disease control rate was significantly lower in the PE cHCC-CC group (58.8%) than in the HCC-control group (86.4%; *P* = 0.01), but the difference was not significant between the GE cHCC-CC (80%) and PE cHCC-CC (58.8%) groups (*P* = 0.17).

**Fig 2:**
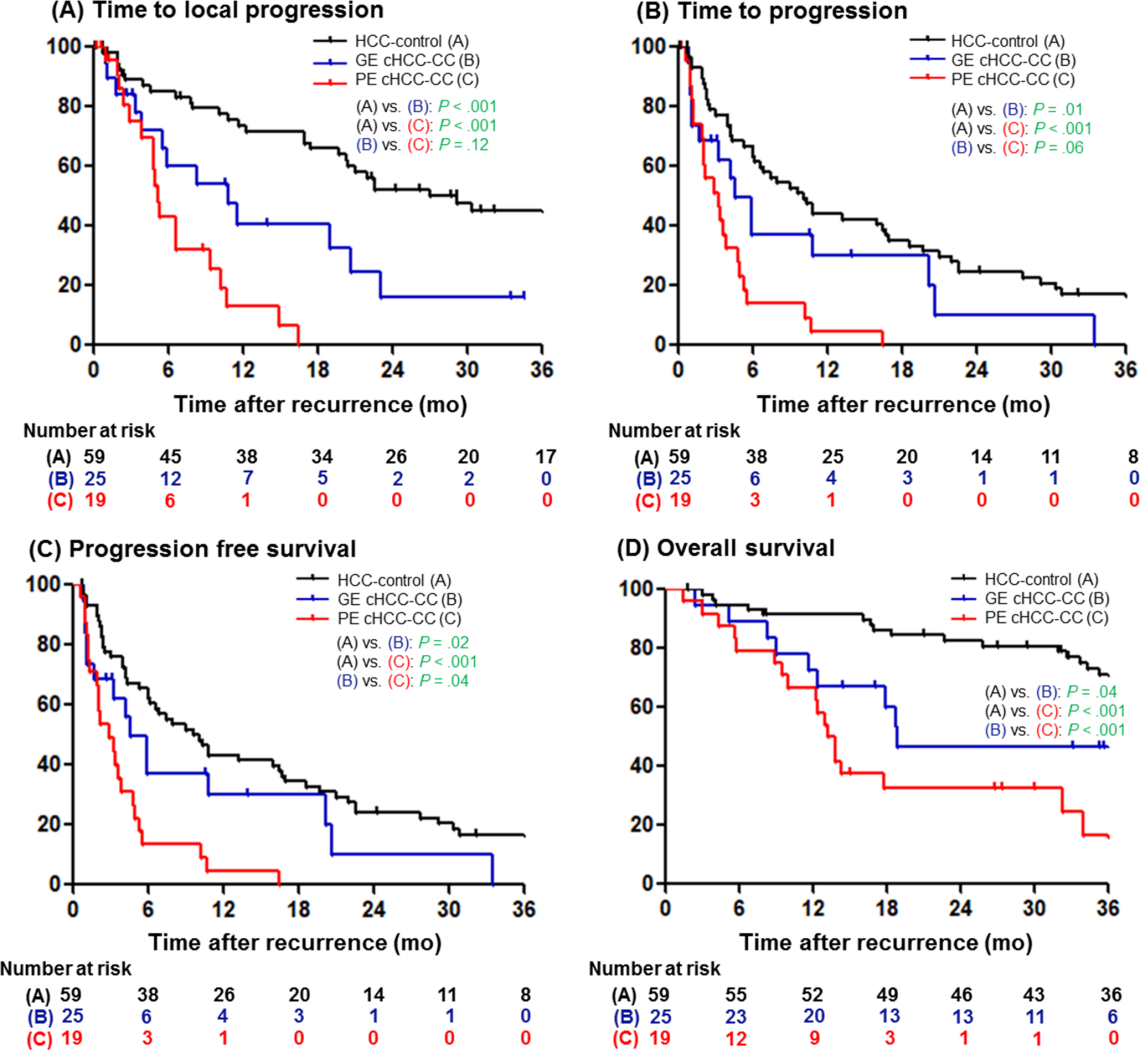
Kaplan-Meier curves of GE cHCC-CC, PE cHCC-CC and HCC-control. (A) Time-to-local progression, (B) time-to-progression, (C) progression-free-survival and (D) overall survival. cHCC-CC, combined hepatocellular-cholangiocarcinoma; GE, globally enhancing; PE, peripherally enhancing; HCC, hepatocellular carcinoma.

During the follow-up period, tumor progression was confirmed in 37 (88.1%) patients in the cHCC-CC groups. Among these, 13 (30.9%) patients showed progression of the index lesion, 18 (42.9%) patients showed a new hepatic or extrahepatic lesion without progression of the hepatic index lesion, and 6 (14.3%) patients showed a new hepatic or extrahepatic lesion with progression of the hepatic index lesion. To evaluate the effectiveness of TACE on target lesions, the median TTP_local_ was calculated. It was significantly shorter in both the GE cHCC-CC (6.6 months) and PE cHCC-CC groups (5.3 months) than in the HCC-control group (27.1 months, *P* < 0.001; Table 4 and Fig 2A). However, it was not different between the GE cHCC-CC and PE cHCC-CC groups (6.6 vs. 5.3 months, *P* = 0.12). The TTP was also marginally shorter in the PE cHCC-CC group than in the GE cHCC-CC group (2.1 vs 4.7 months, *P* = 0.06) and significantly shorter in the GE cHCC-CC group than in the HCC-control group (10.1 months, *P* = 0.01; Fig 2B).

**Table 4:** Time-to-progression, time-to-local progression, progression-free-survival and overall survival after TACE among groups.

Variables	GE cHCC-CC (n = 25)	PE cHCC-CC (n = 17)	HCC-control (n = 59)	*P*^*a*^	*P*^*b*^	*P*^*c*^
Time to progression	4.7 (2.2–10.3)	2.1 (1.1–3.9)	10.1 (4.0–22.6)	0.06	0.01	<0.001
Time to local progression	6.6 (4.8–19)	5.3 (3.4–10.8)	27.1 (11.7–NR)	0.12	<0.001	<0.001
Progression free survival	4.7 (2.2–10.3)	2.1 (1.2–3.9)	9.7 (4.0–22.6)	0.04	0.02	<0.001
Overall survival after recurrence	52.8 (12.4–NR)	12.4 (5.7–17.8)	67.5 (33.8–NR)	<0.001	0.04	<0.001

Data presented as median months and IQR.

NR, not reached

a, between GE cHCC-CC and PE cHCC-CC

b, between GE cHCC-CC and HCC-control

c, between PE cHCC-CC and HCC-control

### Survival outcome after TACE

The median follow-up duration was 2.0 (IQR, 1.3–3.7) years in the cHCC-CC group and 7.9 (IQR, 3.5–9.1) years in the HCC-control group. During follow-up, 56 (55.4%) patients died (69.0% in the cHCC-CC group and 45.8% in the HCC-control group), mostly as a result of cancer progression. The PFS of the PE cHCC-CC, GE cHCC-CC, and HCC-control groups was significantly different (2.1 vs 4.7 vs 9.7 months; *P* = 0.04 for GE cHCC-CC vs PE cHCC-CC, *P* = 0.02 for GE cHCC-CC vs HCC-control group, and *P* < 0.001 for PE cHCC-CC vs HCC-control; Table 3 and Fig 2C).

As shown in Table 4 and Fig 2D, there were significant differences in OS between the three groups (12.4 months in the PE cHCC-CC group, 52.8 months in the GE cHCC-CC group, and 67.5 months in the HCC-control group; *P* < 0.001 for GE cHCC-CC vs PE cHCC-CC and *P* = 0.04 for GE cHCC-CC vs HCC-control).

### Predictive factors for poor outcomes after TACE in patients with recurrent cHCC-CC after surgical resection

We performed Cox regression analyzes for TTP_local_ and OS. Through univariable analysis, PE cHCC-CC at the time of initial diagnosis, PE cHCC-CC at the time of recurrence, multiple recurrences, and maximum diameter of the recurrent tumor were selected as variables for multivariable analysis. After subsequent multivariable Cox analysis using the backward elimination method, the adjusted HRs for TTP_local_ revealed an independent association with the enhancement pattern of recurrent cHCC-CC at the time of recurrence (HR 2.46; 95% CI 1.10–5.46; *P* = 0.03; Table 5). Other predictors related to short TTP_local_ were multiple recurrences and maximum tumor diameter (HRs 5.13 and 1.04, respectively; *P* < 0.05 for each).

**Table 5:** Predictive factors of TTP_local_ and overall survival after TACE in recurred cHCC-CC patients.

	Time-to-local progression*			
**Variables**	**Univariable analysis**		**Multivariable analysis**	
	**HR (95% CI)**	***P***	**HR (95% CI)**	***P***
PE cHCC-CC (initial)	2.48 (1.12–5.52)	0.03		
PE cHCC-CC (recur)	1.82 (0.85–3.90)	0.12	2.46 (1.1–5.46)	0.03
Child-Pugh class B (recur)	1.69 (0.22–12.80)	0.61		
Multiple tumors (recur)	3.50 (1.53–7.98)	0.003	5.13 (2.1–12.53)	<0.001
Maximal tumor diameter (recur)	1.02 (1.00–1.04)	0.03	1.04 (1.02–1.07)	0.002
Gross vascular invasion (recur)	0.90 (0.31–2.61)	0.84		
Distant metastasis (recur)	2.14 (0.83–5.54)	0.12		
	Overall survival*				
**Variables**	**Univariable analysis**			**Multivariable analysis**	
	**HR (95% CI)**	***P***		**HR (95% CI)**	***P***
PE cHCC-CC (initial)	2.21 (1.00–4.89)	0.051		
PE cHCC-CC (recur)	3.77 (1.71–8.30)	0.001	5.97 (2.38–14.96)	<0.001
Age (recur)	0.97 (0.94–1.00)	0.06	0.95 (0.91–0.99)	0.03
Male sex	1.06 (0.40–2.81)	0.91		
Child-Pugh class B (recur)	11.29 (2.26–56.37)	0.003	45.92 (6.58–320.35)	<0.001
Multiple tumors (recur)	1.93 (0.82–4.55)	0.13		
Maximal tumor diameter (recur)	1.02 (1.00–1.05)	0.04	1.04 (1.01–1.07)	0.009
Gross vascular invasion (recur)	1.18 (0.35–3.94)	0.79		
Distant metastasis (recur)	5.35 (2.19–13.06)	<0.001	7.93 (2.85–22.06)	<0.001

HR, hazard ratio; CI, confidence interval; PD, progressive disease.

*Variables that had a P value of less than 0.1 in univariate analysis were included in subsequent multivariable analysis.

Likewise, the enhancement pattern of cHCC-CC at the time of recurrence was independently related to OS. Through univariable analysis, PE cHCC-CC at the time of initial diagnosis, PE cHCC-CC at the time of recurrence, age at the time of recurrence, Child-Pugh class B, multiple recurrences, maximum diameter of the recurrent tumor, and distant metastasis were selected as variables for multivariable analysis. Unadjusted and adjusted HRs of PE cHCC-CC on OS were respectively 3.77 (95% CI 1.71–8.30; *P* = 0.001) and 5.97 (95% CI 2.38–14.96; *P* < 0.001; Table 5). Other covariates independently associated with OS were age, Child-Turcott-Pugh class, maximum tumor diameter, and presence of distant metastasis (HRs 0.95, 45.92, 1.04, and 7.93, respectively; *P*s < 0.05).

## Discussion

In the present study, we evaluated the response and prognosis of post-operative recurrent cHCC-CC treated with TACE. We found that the GE cHCC-CC group showed better prognosis and response to TACE than the PE cHCC-CC group, but poorer response and prognosis than the HCC-control group. Interestingly, the enhancement pattern of recurrent cHCC-CC was an independent prognostic factor for both PFS and OS after TACE, but that of pre-operative cHCC-CC was not.

This is the first study to systematically evaluate the therapeutic efficacy of TACE and prognosis for recurrent cHCC-CC compared to those for HCC. There is one report examining TACE in cHCC-CC. Kim et al. reported that vascularity was highly associated with tumor response and survival outcome (HR 4.19) [18]. Recent studies have observed different prognoses according to image patterns of cHCC-CC patients treated with surgery. Mao et al. reported better prognosis of GE cHCC-CC patients [19]. Park at el. also revealed that the enhancement patterns reflect the proportion of tumor components and amount of fibrotic stroma, and demonstrated better prognosis of hypervascular cHCC-CC on gadoxetic acid-enhancement MRI [8]. However, these studies examined patients upon diagnosis and did not describe post-surgical recurrence pattern or treatment efficacy.

Two factors might have influenced the different tumor response and survival among the three groups. First, tumor vascularity may be a determining factor for the response to TACE. In concordance with this scenario, the best objective response to TACE, which reflects the size reduction of the tumor after TACE, was significantly better in the GE cHCC-CC and HCC-control groups than in the PE cHCC-CC group, but was not different between the GE cHCC-CC and HCC-control groups. However, different tumor biology among the three groups might also be an important factor affecting tumor progression and OS. TTP was significantly shorter in the cHCC-CC groups, especially the PE cHCC-CC group, than in the HCC-control group, and 48.6% (18/37) of patients in the cHCC-CC groups had new intrahepatic and/or extrahepatic lesions prior to progression of the index hepatic lesion, suggesting a more aggressive metastatic potential in cHCC-CC.

However, the tumor response to TACE *per se* also seemed to be poorer in the cHCC-CC groups, since the TTP_local_ of the GE cHCC-CC group was significantly shorter than that of the HCC-control group, despite the similar objective response rate. These findings imply that local tumor progression was more rapid in non-responders and/or the response was not durable even in responders in the GE cHCC-CC group. In fact, at the time of recurrence, the cHCC-CC groups showed more multiple recurrences and vascular invasion than the HCC-control group. Moreover, time-to-recurrence after resection in the cHCC-CC groups was shorter than that in the HCC-control group. All these findings suggest that cHCC-CC, especially PE cHCC-CC, has more aggressive tumor biology than HCC.

It should be noted that, in interpreting these results, this study did not reflect the prognosis of all cHCC-CC patients who underwent surgery. To evaluate the efficacy of TACE in patients with recurrent cHCC-CC, this study included only cHCC-CC patients who received TACE after recurrence. However, the 1-, 3-, and 5-year survival rates in this study were 88.1%, 41.2% and 30.9%, respectively, which are comparable to the results of a previous study conducted in a more inclusive cohort (73.3%, 35.6% and 30.5%, respectively) [13].

In our study, agreement of image patterns between the primary and recurrent tumors was over 50% in the cHCC-CC groups, and primary hypervascular tumors were more likely to have the same image patterns in the recurrent tumor than PE tumors (77.8% vs. 54.2%). This is concordant with a previous study which also showed over 50% agreement in image patterns [3]. Interestingly, in the GE cHCC-CC group, patients with initial GE and subsequent recurrent GE cHCC-CC showed significantly longer TTP_local_ than patients with initial PE and recurrent GE cHCC-CC (median TTP_local_, 19.0 ± 9.8 vs. 5.4 ± 0.4 months, *P* = 0.01, data not shown).

In our study, a quite number (10/42, 23.8%) of patients with cHCC-CC underwent TACE even in the presence of metastasis. Five patients had lung metastasis, 4 had lymph node metastasis, and one had bone metastasis. Three of these patients had less than 1 cm of lung metastasis and 3 of the lymph node metastases were difficult to diagnose as definite metastasis at the time of TACE.

Our study suggest that TACE is considered to be ineffective in recurred PE cHCC-CC patients and other treatment strategies are needed for these patients. However, there is no established treatment for primary cHCC-CC as well as recurred cHCC-CC because of rarity of patients. We suggest to treat PE cHCC-CC according to cholangiocarcinoma guideline[24]. If recurred PE-cHCC is localized disease, re-operation or radiation therapy could be considered first [25, 26]. If these local modality is difficult, systemic therapy could be considered such as gemcitabine or fluoropyrimidine-based chemotherapy. Combination therapy with immunotherapy and chemotherapy or radiation therapy could be an alternative to the disease with a dismal prognosis.

The present study has some limitations. First, this is a single centre retrospective study with a small population. The rarity of cHCC-CC has been an obstacle in previous studies and will make a larger prospective study difficult. Second, only recurrent cHCC-CCs after surgical resection were included. This was because the diagnosis of cHCC-CC is often difficult with needle biopsy only. As cases proven with needle biopsy only might not represent the general cHCC-CC population, we decided to include recurrent cases only after surgical resection. Third, although we discovered that the enhancement pattern at the time of recurrence was more strongly related to the response to TACE and overall survival than that before surgery, the pathology of recurrent tumors was not confirmed. Moreover, this study did not use the 2010 WHO classification system that categorized cHCC-CCs into classical type and subtypes with stem cell features [27], but the traditional classification system described by Allen and Lisa that categorizes cHCC-CC into double tumor, combined type, and mixed type [28]. Our institution used the traditional classification until 2012, and most of the enrolled patients underwent surgery before 2012. Additionally, the clinical and prognostic implications of the 2010 WHO classification system are still controversial [29–32].

In conclusion, the PE cHCC-CC group showed poorer response and prognosis than the GE cHCC-CC group after TACE. The GE cHCC-CC group showed comparable response to the HCC-control group, although the GE cHCC-CC group had poorer prognosis. Our results suggest that different treatment plans are needed according to enhancement patterns of recurrent cHCC-CC.

## Supporting information

S1 Dataset (XLS)

## Abbreviations

CC: Cholangiocarcinoma
cHCC-CC: combined hepatocellular-cholangiocarcinoma
CI: confidence interval
CR: complete remission
CT: computed tomography
GE: globally enhancing
HCC: hepatocellular carcinoma
HR: hazard ratio
MRI: magnetic resonance imaging
OS: overall survival
PD: progressive disease
PE: peripherally enhancing
PFS: progression-free-survival
PR: partial response
RECIST: Response Evaluation Criteria in Solid Tumors
SD: stable disease
TACE: transarterial chemoembolization
TTP: time-to-progression
TTP_local_: time-to-local-progression
